# Podophyllotoxin Exposure Causes Spindle Defects and DNA Damage-Induced Apoptosis in Mouse Fertilized Oocytes and Early Embryos

**DOI:** 10.3389/fcell.2020.600521

**Published:** 2020-11-19

**Authors:** Lin-Lin Hu, Bi-Yun Liao, Jing-Xi Wei, Yan-Lan Ling, Yu-Xia Wei, Zhong-Lin Liu, Xiao-Qiong Luo, Jun-Li Wang

**Affiliations:** Reproductive Medicine Center, The Affiliated Hospital of Youjiang Medical University for Nationalities, Baise, China

**Keywords:** oocyte, embryo, spindle, oxidative stress, DNA damage, apoptosis

## Abstract

Podophyllotoxin (PPT) is a kind of lignans extracted from the roots and stems of the genus *Podophyllum* from the tiller family, and it has been widely used in the treatment of condyloma acuminatum, multiple superficial epithelioma in the clinics. However, PPT has been reported to be toxic and can cause liver defects and other organ poisoning. In addition, emerging evidences also indicate that PPT has reproductive toxicity and causes female reproduction disorders. In this study, we used fertilized oocytes and tried to explore the effects of PPT on the early embryonic development with the mouse model. The results showed that exposure to PPT had negative effects on the cleavage of zygotes. Further analysis indicated that PPT could disrupt the organization of spindle and chromosome arrangement at the metaphase of first cleavage. We also found that PPT exposure to the zygotes induced excessive reactive oxygen species (ROS), suggesting the occurrence of oxidative stress. Moreover, in the PPT-exposed embryos, there was positive γH2A.X and Annexin-V signals, indicating that PPT induced embryonic DNA damage and early apoptosis. In conclusion, our results suggested that PPT could affect spindle formation and chromosome alignment during the first cleavage of mouse embryos, and its exposure induced DNA damage-mediated oxidative stress which eventually led to embryonic apoptosis, indicating the toxic effects of PPT on the early embryo development.

## Introduction

The early embryonic development quality of mammals is very important for the successful implantation (Teh et al., 2016). The mammalian embryo development begins with the entry of sperm into oocytes and the formation of fertilized oocytes (Duan et al., 2014). When the sperm enters the oocyte, the oocyte completes the second meiosis and forms a zygote (Teh et al., 2016). Subsequently, the embryo undergoes continuous cleavage to form a morula, and then the embryo differentiates into trophoblast ectoderm and inner cell mass, and finally forms a blastocyst (Marikawa and Alarcon, 2009). During this process, important morphological changes such as cell proliferation, compaction, and blastocyst formation occur (Oestrup et al., 2009). There is high incidence for errors of spindle organization, chromosome segregation during the first cleavage of early embryos, and any error occurs in this cellular process will lead to DNA damage and induce apoptosis, which are fatal to the embryo and cause implantation failure. Until now, low embryo quality is one main problem for the fertility and sterility found in the reproductive medicine centers. For the factors that affect embryo quality, besides the genetic mutation or inheritance defects, environmental pollution and excessive drug exposure now become another main concern due to the tremendous consumption, fast development of industries, and increasing diseases in human society.

Podophyllotoxin (PPT) is a lignan extracted from the roots and stems of Podophyllum genus *Podophyllum* in the Berberiaceae family (Umesha and Basavarajuk, 2014). PPT was first isolated in 1880 (Kumari et al., 2017) and it had been discovered for the toxicity of podophyllin on cell stasis and was later classified as PPT (King and Sullivan, 1946). According to clinical and basic research reports, PPT and its derivatives have anti-HIV, immunomodulatory, insecticidal, anti-rheumatic, anti-malaria, and ichthyoxen activities (Chen et al., 2007). In addition, studies also report that PPT has anti-cancer properties and it has attracted widespread attention (Kamal et al., 2015). PPT has strong cytotoxic activity on various cancer cell lines (Zi et al., 2019) and so does the spindle assembly: it can inhibit tubulin polymerization, which further leads the abnormal spindle to disturb mitosis (Desbene and Giorgi-Renault, 2002). In addition, studies have shown that PPT binds to the colchicine site of tubulin, but the binding direction is slightly different (Ravelli et al., 2004). Due to its high toxicity and poor water solubility, its application as an anticancer drug is limited (Zi et al., 2019). Studies have shown that the poisoning reaction of PPT is manifested as nausea and vomiting; in addition, it can cause skin pain and burns and other adverse reactions (Longstaff and von Krogh, 2001). PPT also has reproductive toxicity: it is shown to inhibit rat sperm maturation and fertility by promoting the apoptosis of epididymal epithelial cells (Xie et al., 2017). For female animals, PPT can affect oocyte maturation by causing porcine oocytes to undergo oxidative stress and apoptosis (Jiang et al., 2020). However, until now, the toxic effects of PPT on early embryo quality have not been reported.

In this study, we used mouse fertilized oocytes as an experimental model to explore the toxic effects of PPT during early embryonic development. Our results found that PPT could cause abnormal or even degradation of the spindle and led to disordered chromosome arrangement during the first cleavage of zygotes, which induced early embryo defects. In addition, we also found that PPT could cause DNA damage, which induced oxidative stress and apoptosis of embryos. Therefore, our data provided the evidence for the toxicity of PPT on embryos.

## Materials and Methods

### Antibodies and Chemicals

Mouse anti-α-tubulin antibody was from the Cell Signaling Technology (Beverly, MA, United States). Alexa Fluor 594 and 488 goat anti-rabbit antibodies were purchased from Invitrogen (Carlsbad, CA, United States). All other chemicals and reagents were from Sigma–Aldrich Corp., unless otherwise stated.

### Embryo Culture and PPT Treatment

The study followed by the guidelines of Animal Research Committee of Youjiang Medical University for Nationalities, and was specifically approved by the Animal Research Ethics Committee of Youjiang Medical University for Nationalities. Sexually matured female ICR mice of 6–8 weeks were intraperitoneally injected with 5 IU of pregnant mare serum gonadotropin (PMSG) (Ningbo Second Hormone Factory, China). After 48 h of injection of PMSG, mice were injected with 5 IU human chorionic gonadotropin (hCG) (Ningbo Second Hormone Factory) and immediately mated with male mice. The zygotes were collected from the ampullae of the oviducts after 16 h of injection of hCG and then treated with 0.1% hyaluronidase for 5 min at 37°C. The zygotes were transferred to fresh M16 medium covered with paraffin oil and cultured at 37°C under 5% CO_2_ atmosphere.

The PPT was dissolved in DMSO (concentrated at 10 mM) and diluted in M16 medium to produce a final concentration of 0.5, 1, and 2 nM, respectively. The concentration was due to the previous studies on the PPT (Jiang et al., 2020). And, the zygotes were continuously exposed to PPT until we collected for analysis.

### Immunofluorescence Staining and Confocal Microscopy

The embryos were immobilized with 4% (w/v) paraformaldehyde in PBS 1 h and then permeabilized with 1% Triton X-100 (in PBS) for 15 min at room temperature, where after blocked by blocking buffer (1% BSA-addition of PBS) 1 h at room temperature. For anti-α-tubulin-FITC and anti-gamaH2A.X staining, embryos were incubated with primary antibodies (anti-α-tubulin-FITC, 1:100; anti-gamnaH2A.X, 1:200) overnight. The embryos were further incubated with corresponding secondary antibodies (Alexa Fluor 594 bound goat anti-rabbit antibody, 1:200) for 1 h at room temperature. Then, the embryos were stained with Hoechst 33342 (10 mg/mL in PBS) for 15 min and samples were mounted on glass slides. Finally, the embryos were examined with a confocal laser-scanning microscope (Zeiss LSM 800 META, Germany). Fluorescence intensity was analyzed by image J software. To avoid errors, the embryos of the treatment and control groups were sealed on a sheet of glass and scanned with the same parameters to standardize different replicates. Average fluorescence intensity per unit area of the target region was calculated using image J. When the fluorescence intensity is counted, the embryos with extremely strong and weak fluorescence intensity are excluded. The average fluorescence intensity of all embryos was used as the final average fluorescence intensity.

### Detection of Reactive Oxygen Species (ROS)

We detected the reactive oxygen species (ROS) levels in embryos by DCFH diacetate (DCFHDA) kit (Beyotime, China). The living embryos were incubated with DCFH-DA (1:800) in fresh M16 medium for 15 min at 37°C in a 5% CO_2_ atmosphere, and then were transferred into preheated fresh M16 medium and washed three times. A fluorescence microscope (Zeiss LSM 800 META, Germany) was adopted to detect ROS fluorescent signals.

### Annexin-V Staining of Embryos

The living embryos were incubated with Annexin-V-FITC (1:10) in a buffer solution (China Norway Zambia Biotechnology Co., Ltd.) for 20 min. A confocal fluorescence microscope (Zeiss LSM 800 META, Germany) was adopted to detect fluorescent signals.

### Real-Time Quantitative PCR

We examined the mRNA expression of apoptosis-related genes by real-time quantitative PCR analysis. We collected 30 embryos for each group after 16 h of culture to extract the total RNA by a Dynabeads mRNA DIRECT kit (Invitrogen Dynal, AS, Norway). The first cDNA was produced by PrimeScript RT Master Mix (Takara, Japan). The expression of genes was determined by a fast-real-time PCR system (ABI Step One Plus) and the ΔΔCT method.

### Statistical Analysis

All experimental data were obtained through at least three repeated experiments with result expression as means ± SEMs. Data are assessed using the GraphPadPrism5 software (GraphPad, San Diego, CA, United States) and *t*-tests were used for statistical comparisons. And a *P*-value < 0.05 was considered significant.

## Results

### Effects of PPT on the Developmental Ability of Early Mouse Embryos

**FIGURE 1:**
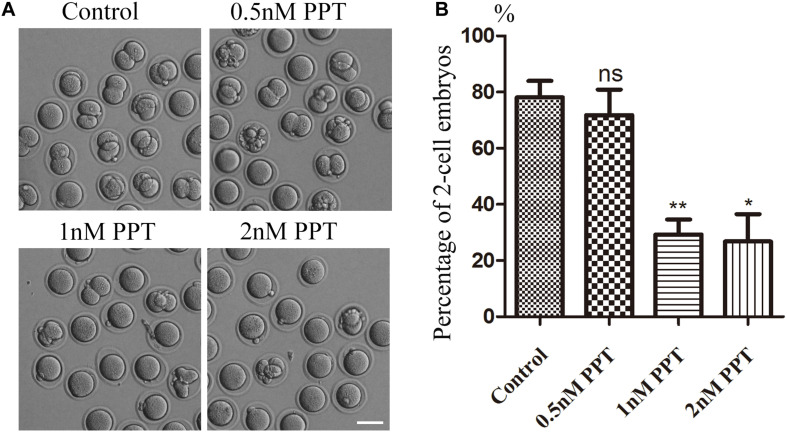
Effects of PPT on the first cleavage of fertilized oocytes in mice. **(A)** Representative images of two-cell embryo formation in the control group and PPT treatment group with different concentrations. Bar = 100 μm. **(B)** Percentage of two-cell embryos in the control and PPT treatment groups with different concentrations. *P < 0.05 and **P < 0.01.

To investigate the toxic effects of PPT on mouse embryos, we first treated fertilized oocytes with different concentrations (0.5, 1, 2 nM) of PPT to observe the developmental status of two-cell embryos. As shown in Figure 1A, most embryos in the control group could reach the stage of two-cell (78.14 ± 5.83%, *n* = 116). There was no significant difference for the 0.5 nM PPT exposed group compared with the control group (71.82 ± 9.05%, *n* = 107, *P* > 0.05). However, in the 1 and 2 nM PPT groups, the percentage of two-cell embryos was significantly decreased compared to the control group (1 nM, 29.29 ± 5.32%, *n* = 120, *P* < 0.01; 2 nM, 26.80 ± 9.76%, *n* = 103, *P* < 0.05) (Figure 1B). Our results suggest that PPT could reduce the first cleavage competence to the two-cell stage in early mouse embryos. 1 nM PPT treatment was used for the following experiments.

### PPT Affects Spindle Morphology and Chromosome Alignment at the First Cleavage of Early Mouse Embryos

**FIGURE 2:**
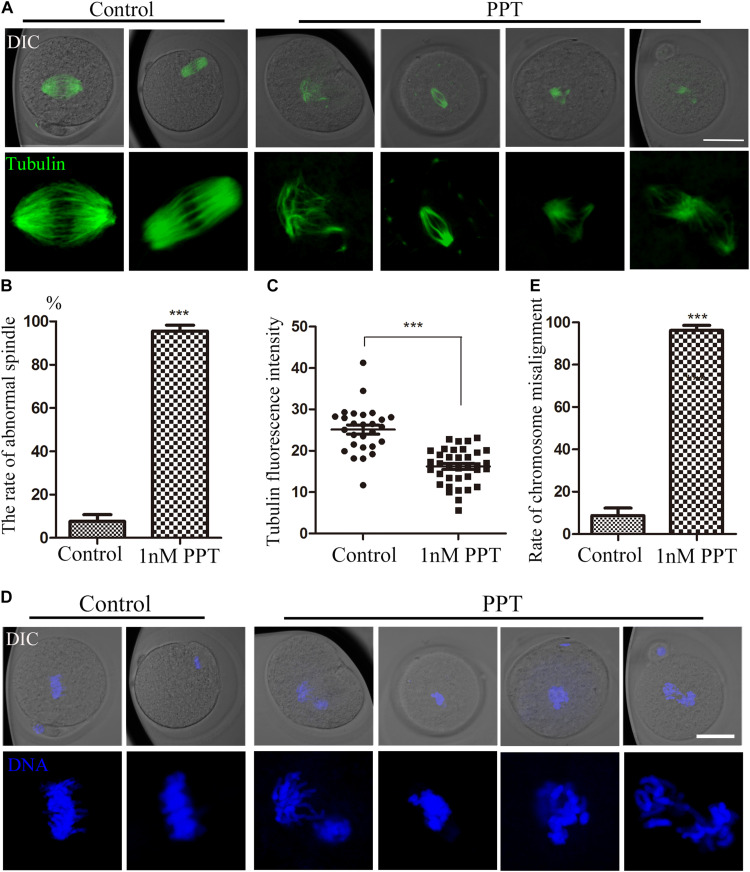
Effects of PPT on spindle morphology and chromosome alignment during the first cleavage of early mouse embryos. **(A)** The typical pictures of spindle morphology after PPT exposure during the first cleavage of early mouse embryos. Bar = 50 μm. **(B)** The rate of abnormal spindle morphology in the first cleavage of mouse embryos after PPT exposure. Compared with the control group, the abnormal rate of spindle in PPT (1 nM) group significantly increased. ***P < 0.001. **(C)** The fluorescence intensity of tubulin after PPT exposure in mice. Compared with the control group, the fluorescence intensity of spindle was significantly reduced in PPT (1 nM) group. ***P < 0.001. **(D)** The typical pictures of chromosome alignment after PPT exposure during the first cleavage of early mouse embryos. Bar = 50 μm. **(E)** The percentage of chromosome misalignment in the first cleavage of mouse embryos after PPT exposure. Compared with the control group, the percentage of chromosome misalignment was significantly increased in PPT (1 nM) group. ***P < 0.001, green, α-tubulin, blue, chromatin. Bar = 50 μm.

For the purpose of exploring how PPT affects early embryo cleavage in mouse, we collected embryos at the metaphase stage of first cleavage to observe the spindle morphology. As shown in Figure 2A, most embryos in the control group had complete spindle organization and chromosome alignment. However, abnormal spindle morphology was observed in the embryos in the 1 nM PPT treatment group, which manifested as spindle disaggregation or multipolar spindle. Next, we calculated the abnormal rate of spindles in each group. The abnormal rate of embryonic spindles in the 1 nM PPT treatment group was significantly higher than that in the control group (95.56 ± 2.72%, *n* = 56, 1 nM PPT; vs 7.58 ± 3.14%, *n* = 39, control, *P* < 0.001; Figure 2B). In addition, the fluorescence intensity of tubulin in the 1 nM PPT group was significantly lower than that of the control group (16.20 ± 0.73, *n* = 36, 1 nM PPT; vs 25.13 ± 1.10, *n* = 27, control, *P* < 0.001; Figure 2C). Moreover, most embryos in the 1 nM PPT treatment group showed severe chromosome arrangement disorders (Figure 2D). And our statistical analysis data also confirmed this: the abnormal chromosomal arrangement rate of embryos in the 1 nM PPT group was significantly higher than that in the control group (96.24 ± 2.37%, *n* = 56, 1 nM PPT; vs 8.69 ± 3.61%, *n* = 36, control, *P* < 0.001; Figure 2E). These results indicate that PPT disaggregates the spindle and destroys the chromosome alignment during early mouse embryonic development.

### PPT Exposure Induces DNA Damage in Early Mouse Embryos

**FIGURE 3:**
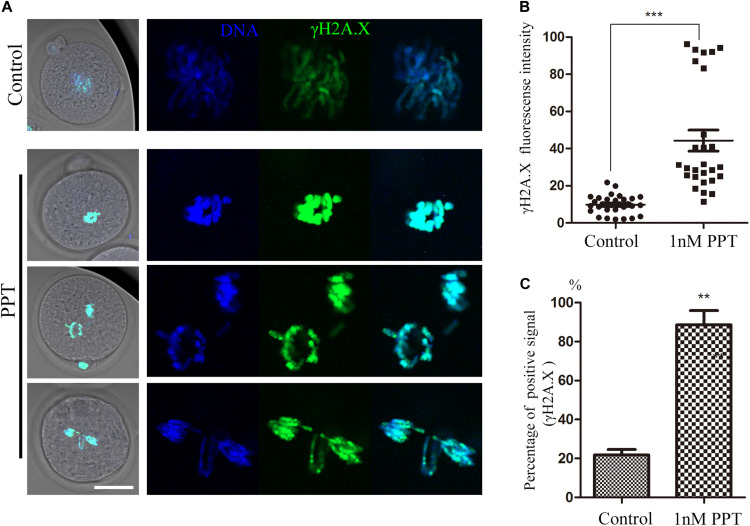
Effects of PPT exposure on the DNA damage of the first cleavage of early mouse embryos. **(A)** Typical pictures of DNA damage in embryos after PPT exposure. Green, γ-H2A.X; blue, chromatin. Bar = 50 μm. **(B)** The fluorescence intensity of γ-H2A.X after PPT exposure in mice. Compared with the control group, the fluorescence intensity of γ-H2A.X was significantly increased in PPT (1 nM) group. ***P < 0.001. **(C)** Percentage of γ-H2A.X positive signals after PPT exposure. Compared with the control group, the percentage of γ-H2A.X positive signals was significantly increased in PPT (1 nM) group. **P < 0.01.

In order to further explore the toxic mechanism of PPT on the early embryonic development of mice, we used γH2A.X to detect the effect of PPT exposure on the DNA damage during the first cleavage of the embryos. As shown in Figure 3A, the immunofluorescence results showed that compared with the control embryos, there were strong positive signals of γH2A.X on the chromosomes of embryos in the 1 nM PPT group. The analysis of fluorescence intensity confirmed that the γH2A.X in the 1 nM PPT group was significantly higher than that in the control group (44.31 ± 5.66, *n* = 27, 1 nM PPT; vs 9.88 ± 0.92, *n* = 30, control, *P* < 0.001; Figure 3B). In addition, the percentage of embryos with the positive γH2A.X signals in the 1 nM PPT group was also significantly higher than that of the control group (88.64 ± 7.31, *n* = 34, 1 nM PPT; vs 21.82 ± 2.78, *n* = 32, control, *P* < 0.01; Figure 3C). These results suggest that PPT could cause DNA damage in mouse embryos during early cleavage.

### PPT Exposure Induces Oxidative Stress in Early Mouse Embryos

**FIGURE 4:**
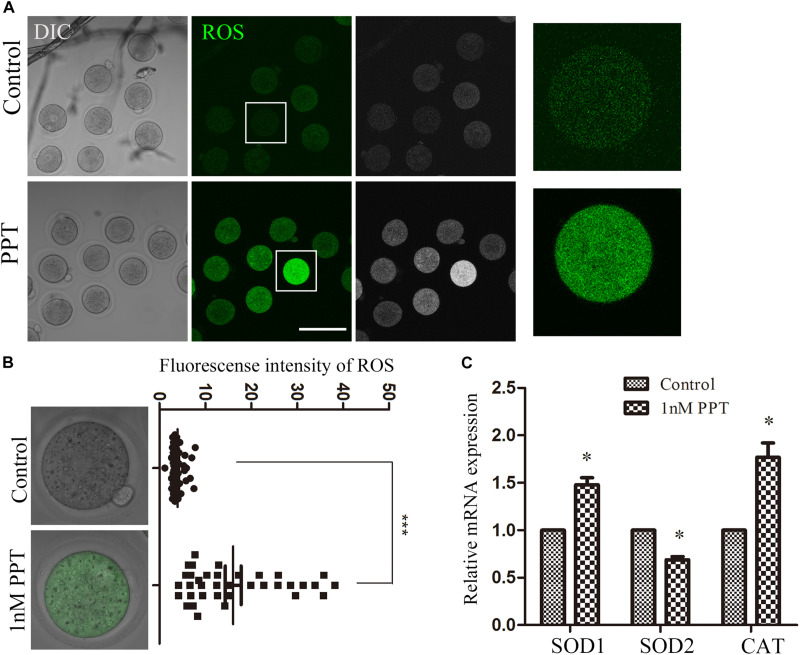
Effects of PPT exposure on oxidative stress of the first cleavage of early mouse embryos. **(A)** Typical pictures of the ROS level in the embryos after PPT exposure. Bar = 150 μm. **(B)** The relative fluorescence intensity of ROS after PPT exposure in mice. Compared with the control group, the DCHF-DA fluorescence (green) of the embryos was significantly increased in PPT (1 nM) group. ***P < 0.001. **(C)** The expression of ROS-related genes in the PPT (1 nM) group and control group. ^∗^*P* < 0.05.

Since previous studies showed that DNA damage increases the level of ROS in somatic cells, we next investigated whether PPT exposure could induce oxidative stress in early mouse embryos. We collected embryos in the first cleavage stage and stained them with DCFH-DA to explore the alteration of ROS level. The results of fluorescence staining showed that the ROS level of the PPT-exposed group was significantly higher than that of the control group (Figure 4A). The fluorescence intensity analysis of ROS also confirmed this point: as shown in Figure 4B, the fluorescence intensity of embryos in the 1 nM PPT group was significantly higher than that of the control group (16.08 ± 1.74, *n* = 36, 1 nM PPT; vs 3.99 ± 0.22, *n* = 43, control, *P* < 0.001). In addition, we explored the expression of several genes related to oxidative stress through RT-PCR. Compared with the control group, there were significantly increase for the expression of catalase (CAT) (1.00 vs 1.76 ± 0.15) and superoxide dismutase 1 (SOD1) (1.00 vs 1.48 ± 0.007), and a significantly decrease for the expression of superoxide dismutase 2 (SOD2) (1.00 vs 0.68 ± 0.03) in the 1 nM PPT-exposed group (Figure 4C). These results indicate that PPT exposure causes oxidative stress in early mouse embryos.

### PPT Exposure Induces Apoptosis in Early Mouse Embryos

**FIGURE 5:**
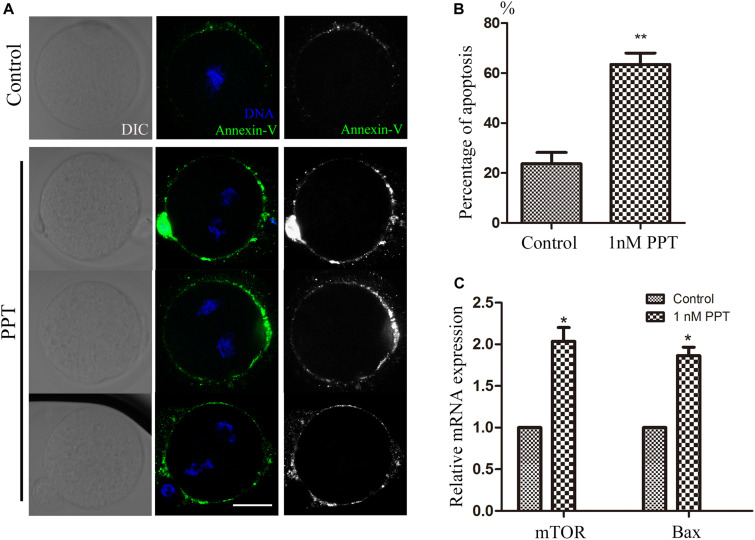
PPT exposure induced apoptosis during early embryonic development in mice. **(A)** Typical pictures of apoptosis in the embryos after PPT exposure. Bar = 30 μm. **(B)** The relative fluorescence intensity of apoptosis after PPT exposure in mice. Compared with the control group, the apoptosis fluorescence (green) of the embryos was significantly increased in PPT (1 nM) group. ^∗∗^*P* < 0.01. **(C)** The expression of apoptosis-related genes in the PPT (1 nM) group and control group. ^∗^*P* < 0.05.

Since oxidative stress generally causes cell apoptosis, we then used live cell staining of Annexin-V to observe whether the embryos in the PPT-exposed group suffered from early apoptosis. As shown in Figure 5A, positive signals of Annexin-V were observed on the cell membrane of embryos in the 1 nM PPT-exposed group, while there were barely signals of Annexin-V in the control group. Compared with the control group, the percentage of positive apoptosis signals in the 1 nM PPT-exposed group was significantly increased (23.72 ± 4.49, *n* = 38, control; vs 63.48 ± 4.53, *n* = 36, 1 nM PPT, *P* < 0.01; Figure 5B). In addition, we explored the expression of several genes related to apoptosis through RT-PCR. Compared with the control group, the 1 nM PPT-exposed group embryos showed significantly increase for the expression of mTOR (1.00 vs 2.03 ± 0.16) and Bax (1.00 vs 1.86 ± 0.10) (Figure 5C). These results indicate that PPT exposure causes early apoptosis in early mouse embryos.

## Discussion

In this study, we explored the toxic effects of PPT exposure on early embryonic development in mice. Our results indicated that PPT was developmentally toxic to early mouse embryos, showing with disrupted spindle morphology and disordered chromosome arrangement during the first cleavage of fertilized oocytes. In addition, PPT could cause DNA damage and oxidative stress, which further induces early apoptosis in the early embryos.

To explore the effects of PPT exposure on early embryo development, we first treated the early embryos with different concentrations of PPT to observe the two-cell development rate. Our results showed that the disturbation on the embryo development increased with the higher PPT concentration. Studies have shown that PPT can affect oocyte maturation by destroying the structure of the spindle (Jiang et al., 2020). Combined with our preliminary results, it suggested that PPT was toxic to both oocyte and embryo development. In order to investigate the causes of abnormal cleavage by PPT exposure, we examined the spindle configuration and chromosome arrangement. The results showed that PPT could cause abnormal spindles in the first cleavage stage of early mouse embryos, as well as abnormal chromosome arrangement. This might be one reason for the reduced ratio of two-cell embryos. Studies have shown that PPT treatment can induce mitotic arrest and pro-apoptotic ER stress in lung cancer (Chen et al., 2013), indicating the disturbance of cell cycle. In addition, PPT acetate promotes cancer cell death through cell cycle arrest, ER stress, and autophagy (Choi et al., 2015). Therefore, our results indicated that PPT could affect the embryo’s first cleavage by causing abnormal spindle configuration and chromosome arrangement.

The endogenous DNA damage caused by DNA demethylation and replication errors in zygotes has been shown to frequently occur (Menezo et al., 2010; Wossidlo et al., 2010), and DNA damage affects the integrity of the embryo genome (Palou et al., 2017). Studies have shown that derivatives of PPT can cause DNA damage and apoptosis in breast cancer cells (Wang et al., 2018). And other toxins, such as zearalenone, can cause DNA damage during early embryogenesis in pigs (Xu et al., 2019). So, we next explored whether PPT caused DNA damage to early embryos in mice. The results showed that PPT exposure could lead to an increase in early embryonic γ-H2A.X in mice, indicating the occurrence of DNA damage in embryos. The effects of other environmental factors on the DNA damage in oocytes were widely reported, for example, HT-2 toxin exposure could induce DNA damage in mouse early embryos (Zhang et al., 2019), and this might be due to the defects of cell cycle factors, such as CHK1, since the disruption of these factors all could induce DNA damage in the early embryos (Ju et al., 2020).

When cells suffer from stress, low levels of ROS are beneficial, which promote adaptation to stress through signaling; however, high levels of ROS are harmful and promote oxidative stress in cells (Scialo et al., 2017). PPT has been shown to cause oxidative stress and apoptosis in porcine oocytes (Jiang et al., 2020). Mycotoxins, such as AFB1, deoxynivalenol, and HT-2 toxins, could also cause oxidative stress in oocytes and other cell types (Yang et al., 2014; Zhang et al., 2016; Lu et al., 2018). In addition, the excessive accumulation of histone H2A variant H2AX in cells undergoing DNA damage can increase the activity of NADP (H) oxidase (NOx), thereby increasing the level of ROS. Therefore, DNA damage can increase ROS levels (Rowe et al., 2008; Kang et al., 2012). Our results suggested that PPT led to the production of ROS in early embryos of mice, and the changes of ROS-related gene expression further confirmed the occurrence of oxidative stress. The environmental effects on the reproduction, especially on oocyte and embryo quality, are mostly reflected through ROS level, for example, MEHP exposure causes increased ROS and oxidative stress in mouse oocytes, which could be rescued by anti-oxidant drug melatonin (Zhang et al., 2018). Apoptosis is a complex process that is responsible for removing damaged cells from living organisms (Kerr et al., 1972). It is a programmed cell death related to the characteristic morphology and biochemical changes of cells (Majtnerova and Rousar, 2018). Studies have shown that 2,2′-,4,4′-tetrabrominated diphenyl ether (PBDE47) can induce oocyte apoptosis by inducing oxidative stress in mouse oocytes (Sun et al., 2020). In addition, studies have shown that Fipronil can cause a significant increase in the level of ROS in porcine oocytes and severe DNA damage, thereby causing cell apoptosis (Zhou et al., 2019). PPT derivative could induce apoptosis, cell cycle arrest, and autophagy in hepatoma HepG2 cells (Ren et al., 2018). And our results suggested that PPT induced oxidative stress in the embryo, which caused early apoptosis, and the changes in the expression of apoptosis-related genes further confirmed the occurrence of apoptosis. Similar results are reported in the deoxynivalenol and T-2 toxin exposure on oocyte and embryos, indicating the general toxic effects of these environmental endocrine disruptors on oocyte/embryo quality (Han et al., 2016; Zhu et al., 2016). Therefore, these data all confirm the toxicity of PPT on the early cleavage of mouse embryos.

## Conclusion

Our results indicated that PPT exposure could destroy the spindle morphology and chromosome arrangement, and it also induced DNA damage-depended oxidative stress and early apoptosis, which led to early embryo cleavage defects in mice.

## Data Availability Statement

The original contributions presented in the study are included in the article/supplementary material. Further inquiries can be directed to the corresponding author/s.

## Ethics Statement

The animal study was reviewed and approved by the Animal Research Ethics Committee of Youjiang Medical University for Nationalities.

## Author Contributions

L-LH and J-LW designed the study. L-LH performed the experiments. B-YL, J-XW, Y-LL, Y-XW, and Z-LL contributed the materials. L-LH and X-QL wrote the manuscript. L-LH, X-QL, and J-LW analyzed the data. All the authors approved the final manuscript.

## Conflict of Interest

The authors declare that the research was conducted in the absence of any commercial or financial relationships that could be construed as a potential conflict of interest.
